# Surgical Outcomes and Prognostic Impact of Lung Cancer Associated with Cystic Airspaces: A Retrospective Analysis of 75 Cases

**DOI:** 10.5761/atcs.oa.25-00070

**Published:** 2026-03-12

**Authors:** Yuichiro Machida, Kento Suzuki, Mitsunobu Ino, Takumi Sonokawa, Norihito Kawasaki, Jitsuo Usuda

**Affiliations:** Department of Thoracic Surgery, Nippon Medical School, Tokyo, Japan

**Keywords:** lung cancer associated with cystic airspace, surgical resection

## Abstract

**Purpose:**

The present study reviewed surgical cases of lung cancer associated with cystic airspace (LCCA) and examined the imaging and clinicopathological features of these cases.

**Methods:**

A total of 75 patients with lung cancer associated with cystic airspace, who underwent lung cancer surgery in our hospital between January 2017 and December 2020, were included. We retrospectively analyzed the association between postoperative recurrence of lung cancer and lung cancer associated with the cystic airspace using the Cox proportional hazards model.

**Results:**

Patients with LCCA had a worse prognosis than those with non-LCCA. Furthermore, a univariate analysis showed a significant difference between sex, smoking, differentiation, tumor size, Stage, and LCCA, while a multivariate analysis showed a significant difference between Stage and LCCA. LCCA cases were classified into four categories, as reported in a previous study. Types I and III showed more adenocarcinomas, while Type IV tended to show squamous cell carcinomas.

**Conclusions:**

LCCA has a poor prognosis. It is often difficult to determine which of the T factors of the TNM classification are applied. Therefore, further studies are needed to accumulate more LCCA cases.

## Introduction

Lung cancer is one of the most well-known malignant tumors and the leading cause of cancer-related death worldwide.^1)^ The widespread use of computed tomography (CT) in lung cancer screening and the evolution of lung cancer screening programs have led to relatively early detection of lung cancer.^2)^ With the widespread use of radiological screening, the detection of cystic air spaces is increasing.^3)^ Lung cancer associated with cystic airspace (LCCA) is often misinterpreted as inflammation or infection,^4)^ and there are few reports of surgery for LCCA.

The present study reviewed surgical cases of LCCA and examined the imaging and clinicopathological features of the disease.

## Materials and Methods

### Patients

We retrospectively reviewed a database of 75 patients diagnosed with LCCA from among 593 patients who underwent surgery for primary lung cancer at Nippon Medical School Hospital between 2017 and 2020. Tumors were classified according to TNM classification (8th edition).^5)^ No benign lesions were found in the resected lesions. This study was approved by the Institutional Review Board of Nippon Medical School, Tokyo, Japan (B-2023-664).

### LCCA classification

**Fig. 1:**
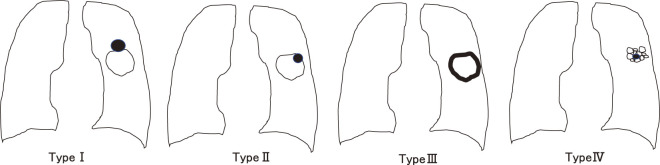
Four classifications of LCCA by Mascalchi et al.^6)^ LCCA: lung cancer associated with cystic airspace

Drawings of the four morphological patterns of LCCA were reported by Mascalchi et al. (**Fig. 1**).^6)^

• Type I: nodule or mass extruded from the wall of the cystic airspace.
• Type II: nodule or mass confined within the cystic airspace.
• Type III: soft tissue density extending along the wall of the cystic air space.
• Type IV: soft tissue density intermixed within clusters of cystic air spaces.

These cases were classified by second thoracic surgeons (YM, TS).

### Statistical analyses

All statistical analyses were performed using the StatView software program (SAS Institute Inc., Cary, NC, USA). Differences between the groups were analyzed using the Pearson *x*^2^ test for categorical variables and an unpaired Student’s *t*-test. Prognostic factors were examined using univariate and multivariate analyses. Disease-free survival (DFS) rates were calculated using the Kaplan-Meier method and compared using the log-rank test. Hazard ratios and confidence intervals (CIs) were estimated using a stratified Cox proportional hazards model. Statistical significance was set at P <0.05.

## Results

### A comparison of LCCA and non-LCCA

The relationship between LCCA and clinicopathological characteristics of patients is shown in **Table 1**. LCCA patients accounted for 10.8% of all who underwent surgery for lung cancer. The median age of patient age was 70.8 (range: 28–91) years old. The LCCA status among the study patients was as follows: LCCA (n = 75, 10.8%) and non-LCCA (n = 518, 89.2%). On fluorodeoxyglucose-positron emission tomography (FDG-PET), the maximum standardized uptake value (SUV_max_) values of LCCA were higher than those of non-LCCA.

**Table 1:** Clinicopathologic characteristics of LCCA and non-LCCA study participants

	LCCA (N = 75)	Non-LCCA (N = 518)	p value
Age	68.6 (28–85)	71.1 (32–91)	0.007
Sex			
Male	59 (79.7%)	290 (56%)	0.003
Female	16 (20.3%)	228 (40%)	
Smoking history			
Ever	70 (93.3%)	351 (67.8%)	<0.001
Never, unknown	5 (6.7%)	167 (32.2%)	
Pathological stage			
Ⅰ	39 (52%)	386 (74.5%)	0.289
II–III	36 (48%)	132 (25.5%)	
Tumor size (invasive diameter)	3.6 (0.8–13.5)	2.4 (0–13.5)	<0.001
FDG-PET			
SUV-Max	8.0 ± 11.2	6.3 ± 6.0	0.478
Differentiation			
G1/G2	46	374	0.072
G3/G4	29	144	
Pathology			
Adenocarcinoma	40 (53.3%)	379 (73.2%)	0.003
Squamous	23 (30.1%)	95 (18.3%)	
Others	12 (16.6%)	44 (8.5%)	
Surgery			
Lobectomy	65 (86.7%)	431 (83.2%)	0.578
Segmentectomy	1 (1.3%)	16 (3.1%)	
Wide wedge resection	9 (12.0%)	71 (13.7%)	

### Prognostic analyses of all cases

**Fig. 2:**
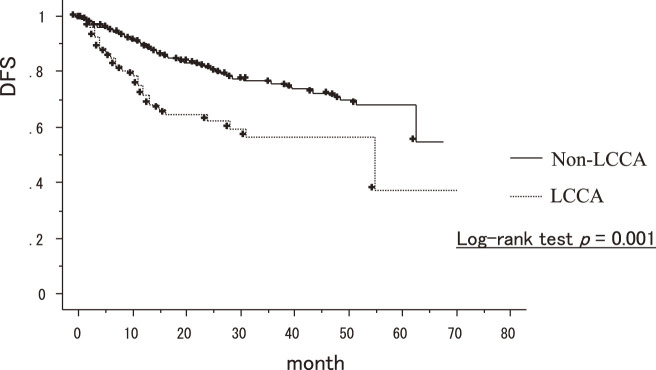
The disease-free survival for LCCA and non-LCCA.

Kaplan–Meier method of disease-free survival showed a significant association between LCCA and non-LCCA (p = 0.001) (**Fig. 2**). Sex (p = 0.0017), smoking (p = 0.016), differentiation (G3/4) (p = 0.0088), tumor size (p <0.001), stage (p <0.001), and LCCA (p = 0.0016) were significantly associated with the DFS in a univariate analysis (**Table 2**). A multivariate analysis of these significant variables showed that LCCA (p = 0.024) and stage (p <0.001) were independently associated with the DFS (**Table 3**).

**Table 2:** A univariate survival analysis examining the association between study participants and the survival

Parameter	HR	95% CI	p value
Gender	0.514	0.338–0.780	0.0017
Age (<75)	1.187	0.811–1.739	0.3778
Smoking	1.758	1.108–2.789	0.0165
Differentiation	0.398	0.199–0.793	0.0088
Tumor size (invasive diameter)	3.961	2.703–5.805	<0.001
Stage	7.502	4.752–10.464	<0.001
LCCA	2.156	1.337–3.478	0.0016

**Table 3:** Results of a multivariate survival analysis examining the association between study participants and the survival

Parameter	HR	95% CI	p value
Gender	1.44	0.843–2.460	0.182
Smoking	1.277	0.281–2.265	0.404
Differentiation	1.942	0.968–3.897	0.062
Tumor size (>3 cm)	0.721	0.443–1.174	0.188
Stage	0.176	0.106–0.290	<0.001
LCCA	0.507	0.281–0.915	0.024

### Consideration of classified LCCAs

The LCCA group was divided according to the classification established by Mascalchi et al. (**Table 4**).^6)^ Types I and III were mostly adenocarcinomas, while Type IV was mostly squamous cell carcinoma.

**Table 4:** Classification of LCCA cases into four types

	I (N = 24)	II (N = 2)	III (N = 23)	IV (N = 26)
Age	66.9 (46–78)	64 (55–73)	67.7 (28–80)	71.2 (48–85)
Sex				
Male	20	1	16	22
Female	4	1	7	4
Smoking history				
Ever	20	2	19	25
Never, unknown	4	0	4	1
Stage				
I	17	0	11	11
II–III	7	2	12	15
Tumor size	2.96 (1.1–11.8)	7.25 (4.5–10)	3.69 (0.8–13.5)	3.38 (1.2–8)
Pathology				
Adenocarcinoma	14	1	19	6
Squamous	5	0	3	15
Others	5	1	1	5
Surgery				
Lobectomy	23	2	21	17
Sublobectomy	0	0	0	1
Wide wedge	1	0	1	7
Others	0	0	1	1

## Discussion

In 1951, the first report of lung cancer associated with bullous lung disease was published by Bass and Singer.^7)^ In 1968, Goldstein et al. described the incidence of bulla-associated carcinoma as 3.8%.^8)^ Hanaoka et al. described the incidence of bulla-associated carcinoma as 3.4%.^9)^ Kaneda et al. reported the incidence of LCCA as 3.5%.^10)^ In our study, LCCA accounted for 10.8% of all cases. We suspect that the rate of LCCA has increased owing to the widespread use of CT in lung cancer screening and increased awareness of LCCA. We believe that the number of LCCA cases will continue to increase even further in the future.

While the pathogenesis of LCCA remains unknown, the check valve mechanism is the most popular theory.^11,12)^ Tan et al.^11)^ observed two types of “check valves”; one type arises from the alveolar wall and consists of tumors producing abundant fibrous tissue, which they reported causes local extrinsic compression of adjacent bronchi that communicate with the cystic vacuole. In the other type, however, tumor cells infiltrate directly into the bronchus or bronchial luminal wall, causing obstruction. The authors explained that these were important inciting events in LCCA. In summary, restricted airflow within the cyst allows microorganisms and carcinogens to deposit on the cyst wall, resulting in recurrent inflammation and formation of a microenvironment for carcinogenesis. Although much remains to be clarified, it is necessary to investigate the pathogenesis of LCCA from a molecular and biological perspective.

There are no clear conclusions regarding the prognosis of LCCA because of the small number of reports. However, based on our results, the prognosis of LCCA is poor, as reported by Jung^3)^ and Kaneda et al.^10)^ By contrast, Hanaoka et al.^9)^ reported that the postoperative survival rate of patients with LCCA is comparable to that of lung cancer patients without LCCA when the disease is resected early. Watanabe et al.^13)^ reported that the thickness of the cavity wall was an independent prognostic factor. Furthermore, Shinohara et al.^14)^ reported that patients with LCCA have a better overall survival rate than those without LCCA. Although the prognosis of LCCA is still under debate, it is necessary to study the prognosis adjusted for the clinicopathologic and imaging features of LCCA in the future.

The LCCA classification was first reported by Meskariki et al.^6)^ They classified lung cancers associated with cystic vacuoles into four morphological patterns, as shown in **Fig. 1**. Types I and II are reported to be associated with a higher frequency of intermediate or poorly differentiated adenocarcinomas than other morphological features on imaging.^6)^ In our study, adenocarcinoma was more common in Types I and III than in other types. This could be a clue to the formation of nodular carcinomas in the cyst wall. Shen et al.^15)^ and Fintelmann et al.^12)^ reported the classification of LCCA, and Shen et al. classified these lesions into four types: Type I, mean wall thickness <2 mm; Type II, mean wall thickness >2 mm; Type III, cystic vacuoles with mural cell nodules; and Type IV, mixed tissue within the cystic vacuole group. A multivariate analysis showed that lung adenocarcinoma with a Type III morphologic pattern was an independent risk factor for high pathologic invasiveness; patients with Type III LCCA had significantly worse survival than those with Type I LCCA. Type III tumors had the highest epidermal growth factor receptor mutation rate.^16)^ Although various classifications have been reported, no classification method has been established. We suggest that it is necessary to create a simpler classification of LCCA in the future.

Several limitations associated with the present study are worth mentioning. The sample size of this study was very small, and the study period duration was a single institution. This was a retrospective study with a relatively short duration. When conducting such a study, it would be desirable to conduct it at multiple institutions with a larger sample size.

## Conclusion

LCCA, especially lung cancers with thick walls or intraluminal nodules, has a poor prognosis. In fact, it is difficult to measure the maximum diameter of a tumor using CT. In addition, it is often difficult to determine which of the T factors of the TNM classification are applied. Therefore, further studies are needed to accumulate more LCCA cases.

## Declarations

### Data availability statement

The data supporting the findings of this study are available within the article and its supplementary materials.
